# Pathways of degradation in rangelands in Northern Tanzania show their loss of resistance, but potential for recovery

**DOI:** 10.1038/s41598-023-29358-6

**Published:** 2023-02-22

**Authors:** Joris H. Wiethase, Rob Critchlow, Charles Foley, Lara Foley, Elliot J. Kinsey, Brenda G. Bergman, Boniface Osujaki, Zawadi Mbwambo, Paul Baran Kirway, Kelly R. Redeker, Susan E. Hartley, Colin M. Beale

**Affiliations:** 1 Department of Biology, University of York, York, YO10 5DD UK; 2 Tanzania Conservation Research Program, Lincoln Park Zoo, Chicago, IL 60614 USA; 3 The Nature Conservancy, Arlington, VA 22203 USA; 4 Tanzania Program, Zoological Society of London, London, NW1 4RY UK; 5 School of Biosciences, University of Sheffield, Sheffield, S10 2TN UK; 6 University of York, York Environmental Sustainability Institute, York, YO10 5DD UK

**Keywords:** Machine learning, Grassland ecology, Environmental impact, Restoration ecology, Computational models

## Abstract

Semiarid rangelands are identified as at high risk of degradation due to anthropogenic pressure and climate change. Through tracking timelines of degradation we aimed to identify whether degradation results from a loss of resistance to environmental shocks, or loss of recovery, both of which are important prerequisites for restoration. Here we combined extensive field surveys with remote sensing data to explore whether long-term changes in grazing potential demonstrate loss of resistance (ability to maintain function despite pressure) or loss of recovery (ability to recover following shocks). To monitor degradation, we created a bare ground index: a measure of grazeable vegetation cover visible in satellite imagery, allowing for machine learning based image classification. We found that locations that ended up the most degraded tended to decline in condition more during years of widespread degradation but maintained their recovery potential. These results suggest that resilience in rangelands is lost through declines in resistance, rather than loss of recovery potential. We show that the long-term rate of degradation correlates negatively with rainfall and positively with human population and livestock density, and conclude that sensitive land and grazing management could enable restoration of degraded landscapes, given their retained ability to recover.

## Introduction

Covering 47% of the terrestrial surface, rangelands are home to one third of the global population, many of whom are pastoralists who depend on rangelands to meet their daily need for shelter, water and food^1–3^. Rangelands are also home to diverse ecosystems, including iconic wilderness areas such as the Serengeti and Ngorongoro. Because rangelands develop in semi-arid areas and are primarily used for grazing, they are often perceived as highly vulnerable to changes in rainfall and anthropogenic pressures^4,5^. With evidence of growing loss and degradation within rangelands and other semi-arid regions, the UN established the Convention to Combat Desertification (UNCCD) in 1996^6^). Although a primary concern that led to the UNCCD were a series of Sahelian droughts that have now ended, concern about loss of rangelands and increasing degradation among the remaining rangeland areas has continued^7,8^). If we are to effectively combat degradation within rangelands, it is important that we understand the drivers of mechanisms by which degradation occurs.

The UNCCD identifies Africa as particularly vulnerable, estimating that land degradation is affecting more than half the continent’s population^9^. Here, rangelands are synonymous with savanna, a biome defined by the presence of C4 grasses, generally occurring in regions with rainfall between 450 mm and 1500 mm per year and often maintained by fire^10–12^. The savanna biome encompasses several habitats, from open grasslands to deciduous woodlands. A key aspect of savanna ecosystems is their high temporal and spatial heterogeneity, a factor that necessitates mobility in human and wildlife populations to exploit patchy resources^13,14^. Societal and land use constraints limit the ability of populations to move when conditions become temporarily unsuitable, and sustained grazing alters the dynamics of savannas, reducing their ability to sustain grazing^15,16^.

Heavy, year-round grazing in savannas reduces grazing potential (i.e. quantity of vegetation palatable to grazers) through two pathways that result in either land invaded by toxic and unpalatable plants, including bush encroachment^8,17^, or in bare ground experiencing soil loss^18^. Rangeland degradation has been defined as a long-term decline in productivity resulting in rangelands unsuitable for grazing (IPBES^19^), rather than short-term declines driven by temporal variability of environmental conditions (e.g. rainfall, grazing pressure). Such degradation has been linked to rainfall patterns^20^, and might be exacerbated by climate-change driven changes in annual rainfall variability, already widely observed across African savannas^21^. At either end of the savanna rainfall gradient, continued precipitation change may interact with pressures like grazing in a way that leads to permanent loss of savannas. Further research into the relationship of these interacting factors with long-term trends of degradation is needed to better understand their importance for sustainable rangeland management.

Degradation and loss of savannas is already a primary driver of poverty and displacement of human populations in Africa. With a rapidly growing human population (averaging a growth rate of 2.2% per year in Africa^22^), the anthropogenic demands on savannas are growing, while the pressures from climate change are simultaneously mounting^3,23^. In a recent study, Hill and Guerschman (2020) identified East Africa as a focal point for increases in bare ground cover, and recommended investigating these trends at a finer spatial scale^24^. In order to meet growing demands from humans and their livestock in the face of potentially deteriorating environmental conditions we must identify how to increase sustainability of savanna use.

Resistance and recovery are two processes that underpin sustainable use of ecological resources^25^, particularly in environments that normally function within cycles of change. Together, these processes define the ’resilience’ of a system^26^. Resistance describes the ability of an ecosystem to continue to maintain function (such as the provision of grazing) despite external pressures, while recovery describes the internal processes that pull a system back towards the pre-disturbed state^26^. In this context, ‘shocks’ are referred to as any event in the environment that leads to reductions in rangeland condition beyond the typical, interannual oscillation around the baseline state of rangeland health. Such events might include extreme droughts, or heavy rain resulting in floods. It is important to distinguish between resistance and recovery because management aimed at increasing recovery might be different to that designed to increase resistance. For example, in rangelands, resistance may be increased by promoting a high diversity of grass species or a particularly beneficial grass community composition^27^, while recovery may require, in addition, temporal variation in grazing pressure, for example through temporary grazing exclusion^28^.

It is unclear whether the recent trend in savanna degradation is driven by reduced recovery potential, a decline in resistance, or both^29^. Observing the long-term trends of rangeland condition, quantified by a degradation index, helps reveal the mechanisms behind eventual degradation of habitats: If degradation is driven by a loss of recovery, areas that become degraded will show the same short-term response (i.e. reduction in rangeland condition) to external shocks as comparable sites, but would be expected to recover more slowly, and potentially insufficiently, before the next shock occurs. Alternatively, if degradation is driven by a loss of resistance, areas that become degraded will show a greater initial response to shocks, and will therefore be less likely to have recovered to pre-shock conditions before another shock occurs, despite similar recovery rates to more resistant areas. Recovery and resistance are not mutually exclusive, and may interact with land management or rainfall conditions to generate different relative impacts in different areas. Quantifying the relative effects of resistance and recovery is important to identify management priorities for savannas^29^.

The rangelands of Northern Tanzania are typical of many African savannas. They are home to significant populations of pastoralists^3,30^ and hold globally important wildlife populations^31^, yet there is widespread concern about their loss and degradation^32^. Wildlife numbers are falling and poverty is high: degradation has been identified as a key contributor to this problem^33^ but is not ubiquitous. Across Northern Tanzanian rangelands there is considerable variation in the degree of degradation and in anthropogenic and environmental drivers of degradation. For example, in our study area, rainfall varies from 400 to 900 mm^34^, human population from 5 to 35 people per km^2^^35^ and livestock densities up to 250 head of cattle per km^2^^36^. These landscape conditions are also moderated by a variety of conservation-related land use restrictions. The combination of all of these interacting components makes Northern Tanzania an ideal location to study the processes that shape recovery and resistance in rangeland dynamics.

Here we combine field data on vegetation structure with high-resolution satellite data gathered over the last two decades (a period spanning two severe droughts) to identify the drivers of degradation within Northern Tanzanian rangelands. Our aims were to (1) test whether long-term changes in grazing potential demonstrate loss of resistance, loss of recovery, or both, and (2) identify how spatial variation in land use designation, human and livestock density, and rainfall patterns impact degradation pathways at a fine spatial scale. We hypothesize that drier areas experience higher rates of degradation, and we expect both livestock and human population density to be positively correlated with degradation. We predict that long-term rates of degradation correspond to the degree to which grazing is managed by official land use designation, with areas that have the most grazing restrictions (i.e. national parks) exhibiting the lowest increases in degradation. Finally, we hypothesize that loss of recovery and loss of resistance both contribute to long term degradation patterns.

## Methods

To test whether loss of resistance or recovery is the primary mechanism driving degradation in Northern Tanzanian rangelands, we (a) chose key parameters that defined degradation within our study area, (b) quantified variation in degradation across the landscape, and (c) evaluated how degradation changed at specific locations over time. We evaluated bare ground cover and the number of invasive & toxic plants (ITP), measured as the abundance of three key plant species, as candidates for degradation parameters. We used field survey data gathered in 2016 and 2018, across sampling sites stratified throughout the study region, to train a machine learning algorithm. Using this algorithm, we estimated degradation parameters for the years 2000 to 2020, based on Landsat satellite images and rainfall data, taking into account seasonal variation of vegetation productivity. Subsets of survey data were used to ground truth and test model outputs. We then tested whether estimated degradation outcomes correlated with spatial maps of anthropogenic and environmental variation. When considering long-term trends in degradation, we accounted for the effects of temporal and spatial variation in annual rainfall.

### Study area

**Figure 1:**
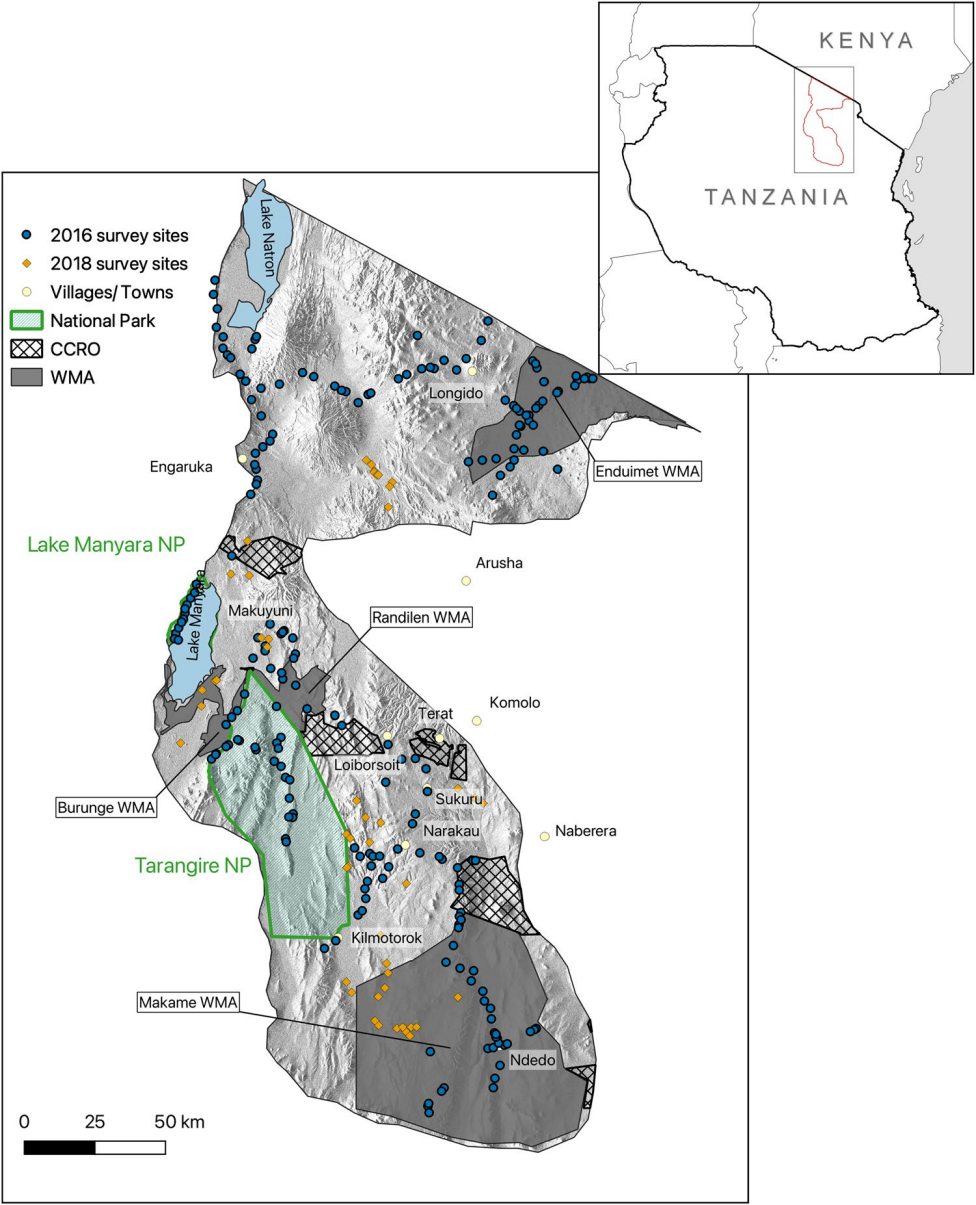
Location of the study area and sites for the baseline vegetation survey in April-May 2016 (blue circles) and April 2018 (orange diamonds). Background shading represents terrain elevation, derived from Shuttle Radar Topography Mission data^48^. Areas not falling into the National Park (NP), Wildlife Management Area (WMA), or Certificate of Customary Right of Occupancy (CCRO) designation were included in the study under the ‘NONE’ category. The map was created using QGIS 3.14^49^.

The study area consisted of 30,300 km^2^ of the Tarangire-Manyara ecosystem and Maasai Steppe of northern Tanzania (Fig. 1). This is a semi-arid ecosystem, dominated by Acacia-Commiphora woodland?. Annual rainfall is bimodal (rains in November to January and March to May) with large inter-annual variability^37^. The 20 years considered in this study covered multiple positive and negative phases of Indian Ocean Dipole (IOD) and the El Niño-Southern Oscillation (ENSO), which represent large scale climate processes that impact rainfall in East Africa^38^. Notable droughts in the study region were recorded in 2003-4 and 2016-17, when average rainfall was around 50% below average (Foley, unpublished data). The period between October 2019 - January 2020 was the wettest recorded in East Africa in over two decades^39^. The stratified sampling locations fell into areas of four different land use strategies. These included, in descending order according to the degree of grazing restrictions:

- NP: Two national parks (Tarangire, established 1970, and Lake Manyara, established 1960, where grazing is outlawed, but some illegal grazing persists^40^.
- WMA: Four wildlife management areas (WMAs) (Enduimet, Randilen, Burunge and Makame), established beginning in 2003^41^. WMAs primarily give communities the rights to manage wildlife on their lands, as well as grazing activities, but all activities are managed by a WMA board.
- CCRO: Areas secured by the Certificate of Customary Right of Occupancy (CCRO) initiative^42^, a relatively new intervention implemented beginning in 2015, whereby communities retain land ownership and decide on land use practices.
- NONE: Land not covered under any official management/protection scheme.

### Choice of degradation parameters

Since this study focused on rangeland habitats, we chose degradation parameters from a grazing potential perspective. We followed the Intergovernmental Science-Policy Platform on Biodiversity and Ecosystem Services (IPBES) definition of rangeland degradation, as “persistent loss of vegetation productivity cover, especially of those plants which support herbivores.”^19^. Our proposed parameters consisted of an index of bare ground cover (absence of grazeable vegetation cover) and the number of individual ITP, which replace plants palatable to herbivores. The latter consisted of three key plant species, all native to the area, but behaving like invasive plants: *Ipomea hildebrantii*, an evergreen woody shrub that can significantly decrease grass biomass production^43,44^; *Solanum campylacanthum*, a thorny shrub which has been shown to be highly toxic to livestock^45^; and *Dichrostachys cinerea*, a fast growing tree species that accounts for the majority of bush encroachment in African savannas^46^.

### Baseline ground surveys

We used ground survey data to train and validate remote sensing estimates of degradation. To select survey sites across the study area (Fig. 1), we stratified the ecosystem to ensure data collection from a complete representative selection of land cover, vegetation quality and rainfall levels. This survey stratification also allowed manageable sampling and route planning for accessing sites across the ecosystem. The strata were based on combinations of rainfall, mean annual NDVI (vegetation greenness) for the year 2015, and land cover type (grassland and woodland) (see section ’Survey site stratification’ for details). We chose April and May as the sampling months since this coincided with the end of the wet season and consequential peak vegetation growth, enabling plant species identification^47^. We randomly selected 250 cells evenly across all strata to sample using a basic vegetation survey, with the centre of each 500 m cell selected as the focal sampling point for the vegetation survey. Neighbouring cells were excluded. Some 43 target cells were unsuitable (e.g. recently cleared crops) or inaccessible, resulting in 208 cells sampled between April and May 2016. To increase the geographic spread of sampling locations, during April 2018 additional 48 vegetation surveys were carried out across the ecosystem, three of which targeted areas containing *D. cinerea*. This allowed us to train the machine learning algorithm on a wider range of values, improving the performance when applied over the full study region. The total number of survey locations used in the analysis was 256.

At each survey site (Fig. 1), we collected data based on the Monitoring Rangeland Health guide^47^, which is designed for rapid vegetation and degradation assessments with minimal equipment. Measurements were taken at sampling points every five meters along four 25 m transects extending north, east, south and west, resulting in a 50 x 50 m cross, diagonally covering a plot of 35 x 35 m. At every sampling point, measurements were recorded at 5 notches along a 1 m measuring stick, leading to a total of 100 individual data points (25 for each of the four 25 m transects). The following measurements were quantified: (1) The number of invasive and/or toxic rangeland species (*I. hildebrantii*, *S. campylacanthum* and *D. cinerea*), as a total count of stems at the survey site across all sampling points. (2) The percentage of bare ground, as the percentage of notches falling onto bare ground, across sampling points at the survey site. Measures of plant density and bare ground were not mutually exclusive, e.g. a count of one plant of *D. cinerea* could coincide with a high percentage of bare ground at a given sampling point, in the absence of ground vegetation.

### Survey site stratification

We used remote sensing data (MODIS), accessed and downloaded using the Google Earth Engine (GEE) cloud computing platform^50^, to stratify vegetation survey locations. This stratification allowed us to collect samples over a range of different environmental conditions. The MODIS products included: MOD13A1^51^, a 16-day Normalized Difference Vegetation Index (NDVI) composite (a measure of vegetation productivity), at a 500 m resolution from which we calculated the mean NDVI value per grid cell from all 2015 data; MCD12Q1^52^, a land cover product providing five annual classification layers for global land cover at a 500m resolution. Land cover per grid cell was calculated at the most common annual BIOME-Biogeochemical Cycles (BGC) classification between 2001 and 2013; MOD09A1^53^, an 8-day land surface reflectance product, downloaded for May 2015-April 2016.We further retrieved rainfall data at 0.05$^{\circ }$ resolution for the years 2000-2020 from CHIRPS version 2^54^, a quasi-global dataset, ranging from 50$^{\circ }$S to 50$^{\circ }$N, that combines satellite imagery with rainfall station data to create a gridded rainfall time series. We calculated mean rainfall for the period 2000-2020, interpolated to the same resolution as the MODIS data. For each 8-day MODIS reflectance tile, we interpolated single missing values in all seven bands due to cloud cover based on the average of the preceding and succeeding tiles. Where there were cells with two or three successive missing values, these were replaced by linear interpolation using the ’na.approx()’ function in the ‘zoo’ package^55^ in R 3.2.2^56^. The sample stratification resulted in a design that spanned a wide rainfall gradient, from 360 mm to 1095 mm total annual rainfall.

### Estimating degradation parameters, using machine learning

We obtained remotely sensed satellite data for the machine learning regression from Landsat through Google Earth Engine, at 30 m resolution, which closely matched the 35 m vegetation survey plots. We combined Landsat 5, 7 and 8 products (courtesy of the U.S. Geological Survey) to maximise data coverage for our study period and region. For Landsat 7 products, the scan line correction device failed in May 2003, leading to a 22% loss in values for each scene^57^. A gap-filling function was applied in GEE to mitigate this. For each Landsat product, we calculated per-pixel cloud scores and only included pixels with less than 10% cloud cover.

Based on the Landsat images, we calculated yearly indices that had the potential to indicate patterns of rangeland degradation at 30 m resolution. All composites were created starting in November the previous year, and ending in October of the given year used for predictions, to capture the seasonality in the region. Vegetation indices are used as a quantitative measure of vegetation productivity; we therefore calculated the enhanced vegetation index (EVI), an index that is optimised for areas with high productivity and variations in soil brightness^58^. Next, we calculated a bare soil index (BSI) based on a formula introduced by Rikimaru et al., which combines the NDVI and normalised difference built-up index^59^, and has been used in similar studies^60^. We calculated the modified soil-adjusted vegetation index (MSAVI), a vegetation index with increased dynamic range over NDVI, and reduced soil background bias^61^. We used harmonic regression to calculate trend variables (magnitude, phase) for EVI, BSI, and MSAVI. This allowed us to capture the seasonal change of vegetation indices (i.e. variation in plant phenologies), a quantification that can improve the predictive power of classifiers^62^, and was lacking from the yearly averages used for the remaining indices. Finally, we included the total yearly rainfall at the pixel level, based on CHIRPS data, to account for the potentially strong effect rainfall might have on the chosen degradation parameters. Recognising that the amount of grass present at the end of a rainy season is influenced both by the severity of the preceding dry season and the total rainfall across the previous rainy season, we computed annual rainfall from May in the previous calendar year, to the end of April in the focal year. Supplementary Fig. S1 online provides a conceptual overview of the steps involved in creating the composite layers. Supplementary Table S1 online gives a summary of all predictor variables used to train the model algorithm. To minimise unnecessary computations, a mask layer was created that excluded any data for non-savanna habitat. Supplementary Table S2 online gives an overview of data products and parameters used to create the mask.

**Figure 2:**
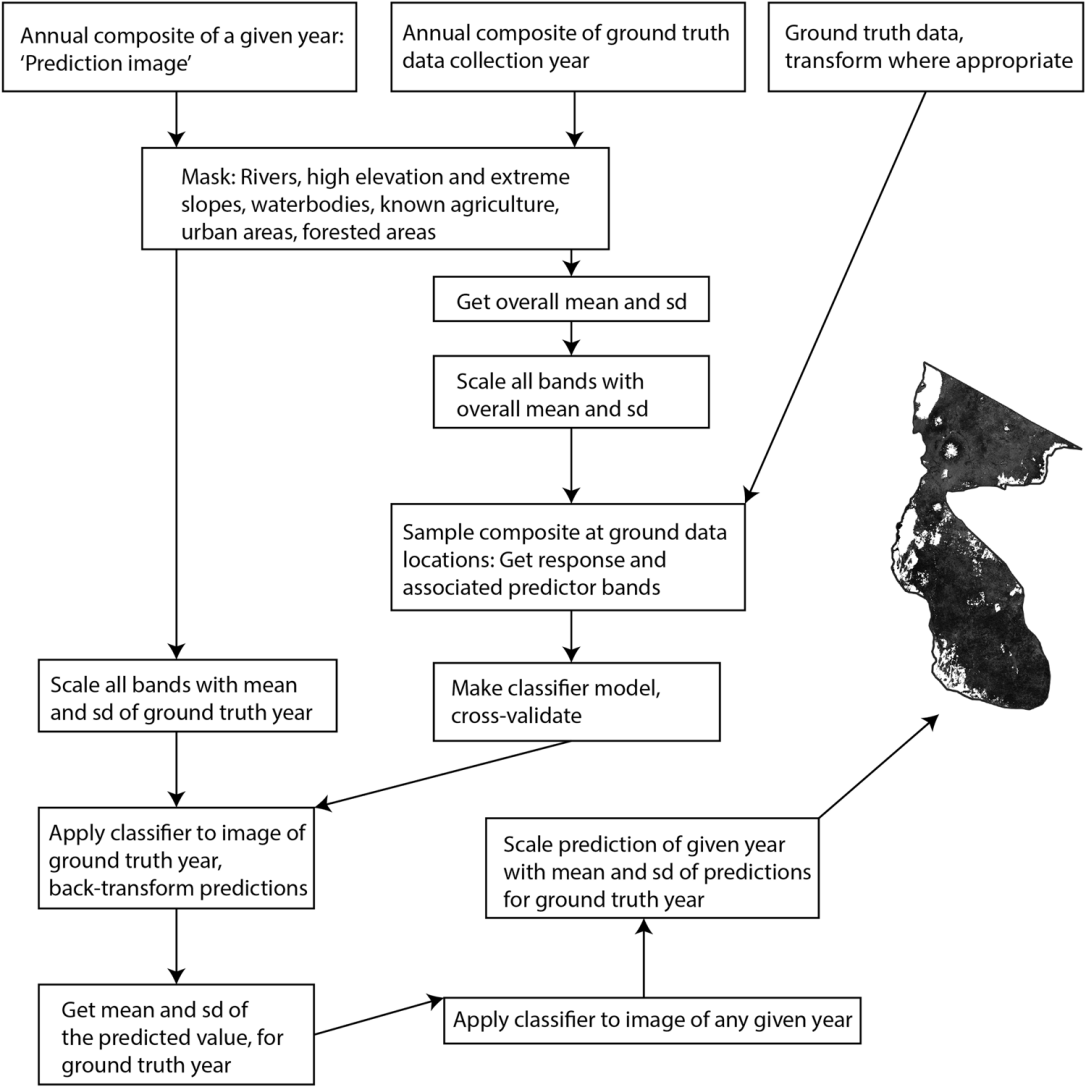
Conceptual overview of the steps involved in creating annual maps of predicted degradation scores for the study area. The map was created using R 3.2.2^56^, and the flowchart was created using Adobe Illustrator Version 23.0.3^69^.

Supervised Machine learning regression algorithms were trained and evaluated in GEE. The surface survey data were joined with predictor variables at the respective sampling locations and years (2016 or 2018), and randomly split into a holdout testing partition (25%) and training partition (75%). The training partition was used for repeated random cross-validation, using ten repeats, and 75%-25% splits for training and validation subsets. Model performance was evaluated using the average r-squared value and RMSE with standard deviation for observed vs. predicted results, across all ten repeats. Model parameters were tuned in GEE, and the final, best performing model was evaluated against the testing holdout partition, previously unseen by the model. This workflow provided us with an unbiased approach to evaluate the models ability to generalize, and avoid overfitting^63^. The predictor variables had very different ranges, and were standardised to improve model performance. The bare ground cover values were skewed towards zero, and an improved model performance was achieved by log-transforming the response: log(x+1). The final model for the classification was trained using all of the ground truth data available. Different classification algorithms were evaluated. Random forest (RF) and support vector regression (SVR) with radial kernel and nu parameter were chosen since they are known to perform well in remote sensing applications^64^. The accuracy performance of one over the other differs between studies^64–66^, warranting a direct comparison. While less often used in remote sensing studies, gradient boosting trees (GBT) have outperformed SVR and RBF algorithms on some occasions^67,68^, and it was therefore included in the comparison. The resulting predictions were back-transformed from the log scale to the original scale for bare ground scores and scaled using the 2016 mean and standard deviation. Final prediction maps were visualised in GEE for every year, to check for abnormalities. Figure 2 gives a conceptual overview of all steps involved in predicting degradation scores, based on the annual composite maps.

### Data analysis

Estimates were made at a 30 m resolution to match the 35 m vegetation plots, but aggregated to 200 m resolution for further analyses using a median. This aggregation reduces stochastic noise potentially arising from extreme values across the 200 m pixels (i.e. very high degradation pixels neighbouring very low degradation pixels), at the cost of ignoring fine-scale patterns. We argue, however, that degradation from a grazing perspective is most meaningful if observed at a scale above 30 m resolution and across the larger scales at which grazing decisions are made. To test hypotheses about resistance and recovery, we normalized degradation scores between 0 and 1, and divided the savanna landscape into three even-sized classes identifying the most, least, and medium degraded areas, based on the median degradation scores of the final three years of the time series (2018-2020). If loss of resistance underpins degradation, we anticipated that sites that are most degraded by the end of the time series would show bigger changes during years with large declines in degradation. If lack of recovery underpins degradation we anticipated that the first year of recovery following a large decline would see smaller recoveries in the most degraded areas. As recovery could be measured in absolute or relative terms (i.e. number of units recovered, or proportion of decline recovered) we considered both quantifications. We used two-way ANOVAs in R to statistically compare classes. We estimated the marginal means for covariates based on the model using the ’emmeans’ package^70^, and conducted Tukey’s post hoc tests for pairwise comparisons. It should be noted that, due to the large sample sizes in these comparisons, traditional statistical significance becomes almost inevitable^71^.

To test hypotheses regarding the mechanisms leading to degradation, we computed the pixel-wise linear rate of degradation parameters at a 200 m resolution across the entire time series (long-term trend), taking into account the annual variation of rainfall when calculating the slope. We fitted a spatially-explicit hierarchical Bayesian regression model using Integrated Nested Laplace Approximation (INLA) with the Stochastic Partial Differential Equation (SPDE) approach in the package ’R-INLA’^72–74^ in R. INLA offers a fast, flexible alternative to Markov-Chain Monte-Carlo methods for fitting complex regression models and allows us to estimate the effects of spatial covariates while accounting for the non-independence of spatial data^75^. The model correlated the long-term rate of degradation to human population density for 2017 (from the Landscan dataset^76^), livestock density for the year 2010 (measured in Tropical Livestock Units based on cattle, goats, and sheep, data from the Gridded Livestock of the World database, FAO^77^), the land use designation (NP, WMA, CCRO, and NONE) as a categorical variable, and total annual rainfall (from the CHIRPS data). Including rainfall as a covariate explicitly accounted for the spatial variation in rainfall, a variable that drives large amounts of variation in grassland productivity^78,79^, and would likely mask trends if unaccounted for. The statistical power of the regression analysis was related to the number of pixels considered. Supplementary Table S3 online gives an overview of the sample sizes used for the land use designation analysis.

Initial models revealed that the estimated range of the spatial autocorrelation was very small, requiring a very fine mesh resolution to fit a smooth SPDE. We created a simple spatial mesh at the point locations using the ’inla.mesh.create’ function in ’INLA’, extending beyond the study region (using the default settings), to avoid boundary effects of the SPDE. We cross-validated the fit of the model by visually inspecting probability integral transform (PIT) values^80^.

## Results

### Model performance

The random forest (RF) model slightly outperformed support vector regression (SVR) for predicting the bare ground scores during cross-validation but performed considerably worse on the holdout partition (Table 1), and SVR was chosen for the final classifier model for the bare ground index. Gradient boosting trees performed best on the holdout partition for predicting the number of invasive & toxic plants (ITP), but considerably worse than SVR and RF during cross-validation. SVR performed better than RF during cross-validation, but performed slightly worse than RF on the holdout partition (Table 1), and RF was chosen for the final classifier model for the number of ITP. See Supplementary Fig. S2 online for validation plots of the final models. When visualizing the prediction maps for the number of ITP, we observed inter-annual variation far greater than plausible for relatively slow-growing woody plants (see Supplementary Fig. S3 online), suggesting that the model did not sufficiently differentiate ITP from the remaining vegetation. We therefore excluded this parameter from further analysis and focused on bare ground cover only.

**Table 1:** Model performance of the different classifiers evaluated for the prediction of a bare ground (BG) and invasive & toxic plants (ITP) index. Values represent averages across 10 random splits of the training partition, with standard deviation. ’Final performance’ represents the performance against the holdout partition.

Classifier	Response	RMSE	R^2^	Final performance
Random Forest	BG	0.16 (± 0.02)	0.41 (± 0.10)	RMSE: 0.17, R^2^: 0.33
	ITP	48 (± 8)	0.53 (± 0.70)	RMSE: 46, R^2^: 0.57
Gradient Boosting Tree	BG	0.21 (± 0.02)	0.38 (± 0.1)	RMSE: 0.20, R^2^: 0.31
	ITP	57 (± 12)	0.42 (± 0.11)	RMSE: 51, R^2^: 0.60
Nu SVR	BG	0.17(± 0.02)	0.38 (± 0.11)	RMSE: 0.15, R^2^: 0.43
	ITP	48 (± 9)	0.59 (± 0.11)	RMSE: 65, R^2^: 0.54

### Spatial patterns

**Figure 3:**
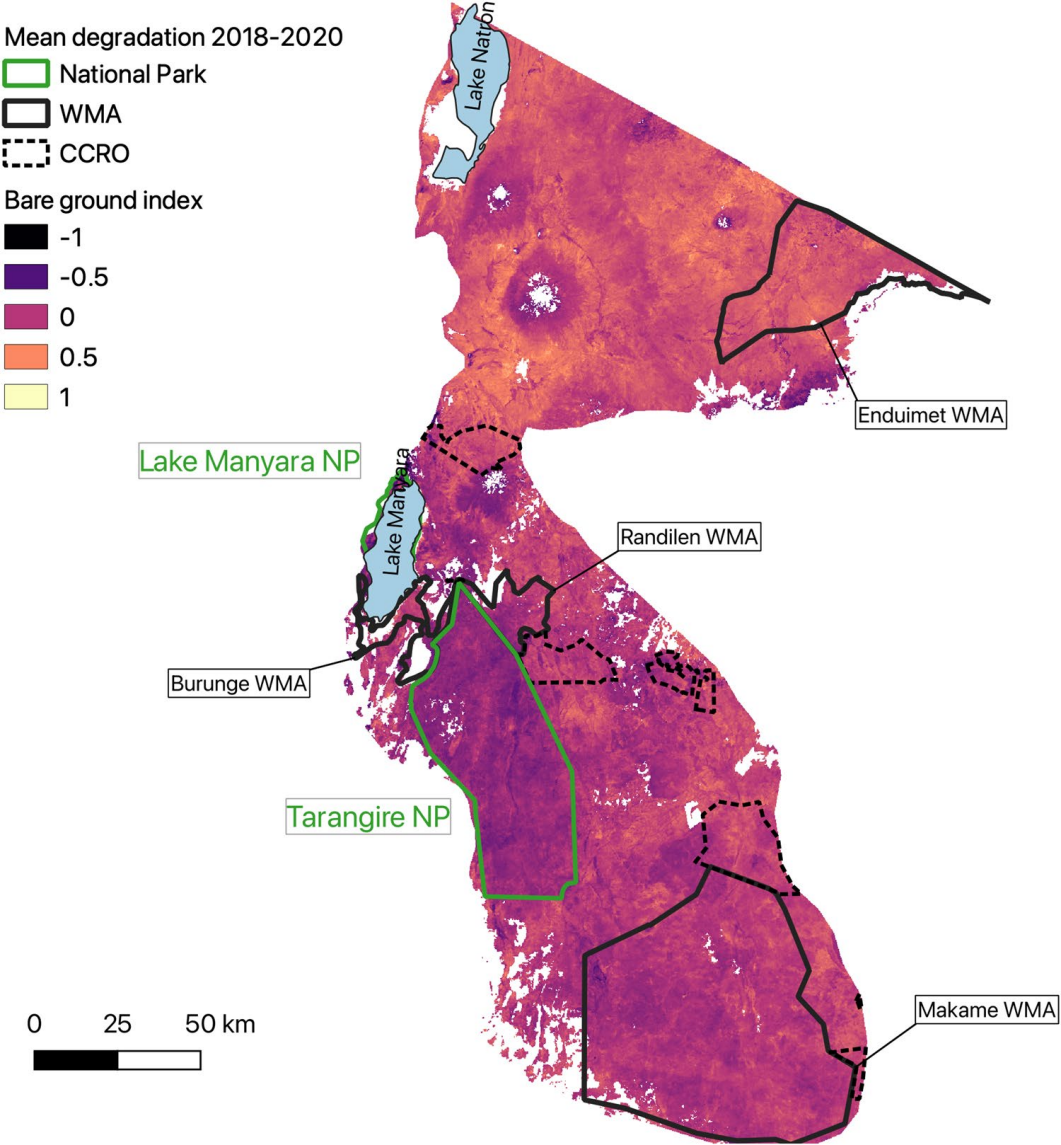
Map of normalized bare ground index scores in the study area, averaged for the years 2018-2020. Areas of high bare ground cover receive higher scores (max index = 1) and are coloured yellow, areas of low bare ground cover have lower scores (max index = -1) and are coloured purple. Gaps in the data (coloured white) are areas removed by the masking layer. The map was created using QGIS 3.14^49^.

Maps of bare ground index scores showed expected spatial patterns. We found lower bare ground cover in Tarangire and Lake Manyara national parks, as well as in the forests surrounding the peaks in the north of the study area (Fig. 3).

### Time series analysis

**Figure 4:**
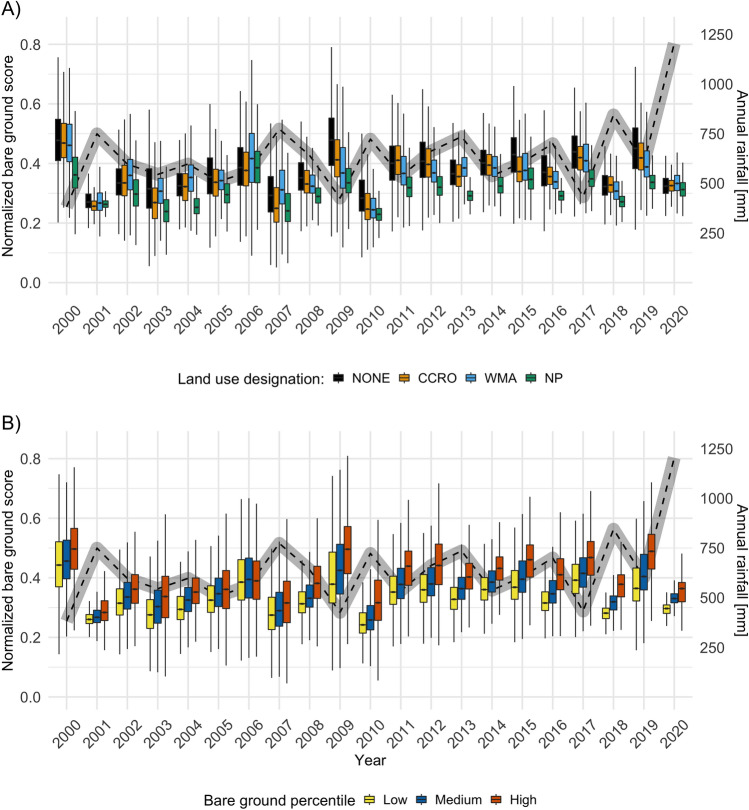
Normalized bare ground index scores over time - areas of high bare ground cover receive higher scores. (**A**) Split by land use designation (black: No official management/protection scheme (NONE), orange: Certificate of Customary Right of Occupancy (CCRO), blue: Wildlife Management Area (WMA), cyan: National Park (NP)) and (**B**) Split by bare ground percentile, based on the median degradation scores of the final three years of the time series (2018-2020). Whiskers represent 95% confidence intervals around the median. Outliers are not shown. The dashed gray line in the background indicates annual median rainfall (November to October), based on CHIRPS version 2 (^54^) data retrieved for the study area.

Annual rainfall in the study area was variable and corresponded to recorded rainfall extremes. During the drought years of 2003-2004, rainfall in the study area remained at a lower level, and rainfall dipped during the drought of 2016-2017 (Fig. 4). The extreme rainfall of 2020 had a strong impact in the study area, with the year exhibiting highest recorded rainfall during the timeline (Fig. 4). We found clear year-to-year variation in bare ground scores (Fig. 4). Consistent land use designation effects were visible throughout the timeline 4A). In most years, national parks showed lower bare ground scores than any other land use designation, with notable exceptions in years of high rainfall (e.g. 2001, 2020). Land that would eventually become CCROs or WMAs often had similarly high bare ground scores to land in the ‘NONE’ category 4A). Areas that were classified as having most, least and medium bare ground scores at the end of the timeline maintained the same classification throughout the study period (Fig. 4B). Recovery years were frequently associated with high annual rainfall (eg. 2001, 2018, 2020) (Fig. 4).

**Figure 5:**
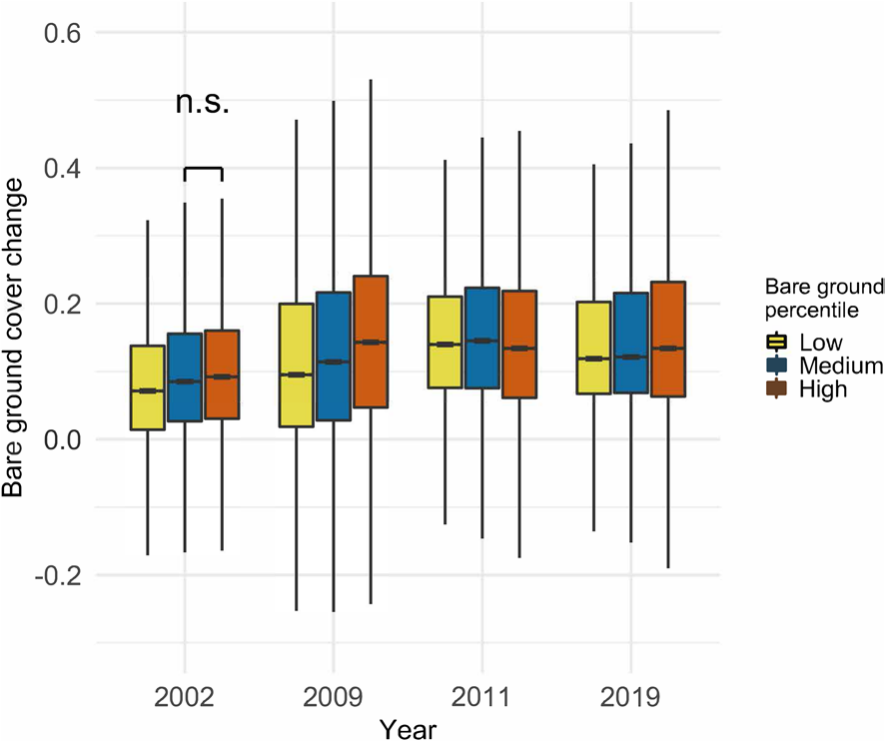
Changes in normalized bare ground index scores during the four years of greatest increase in bare ground cover, Split by bare ground percentile, based on the median degradation scores of the final three years of the time series (2018–2020). Positive numbers along the y axis signify an increase in bare ground cover, while negative numbers signify a decrease. Whiskers represent 95% confidence intervals around the median. Outliers are not shown. All contrasts were statistically different in the ANOVA tests, unless otherwise indicated. n.s.: not significant.

To assess resistance, we focused on the four years with the largest increase in bare ground scores (2002, 2009, 2011 and 2019). We found that cells in the high bare ground percentile increased in bare ground cover more, on average, than other cells in three of the four years assessed (Fig. 5). Similarly, lowest bare ground percentiles in 2018–2020 also showed the lowest average increase in bare ground in three of the four years (Fig. 5).

Looking at the recovery year following large declines, we found that cells ending in the highest bare ground percentile exhibited lower absolute and relative recovery than other cells in the driest recovery year (2003) 6). In the remaining years, cells in the ultimately highest bare ground percentile showed higher absolute recovery than other cells, and the difference increased with higher annual rainfall (6A). In terms of relative recovery, cells in the ultimately highest bare ground percentile showed the same trend of higher recovery in wetter years, and were the only cells with median net improvement in the wettest year (2020) (6B).

**Figure 6:**
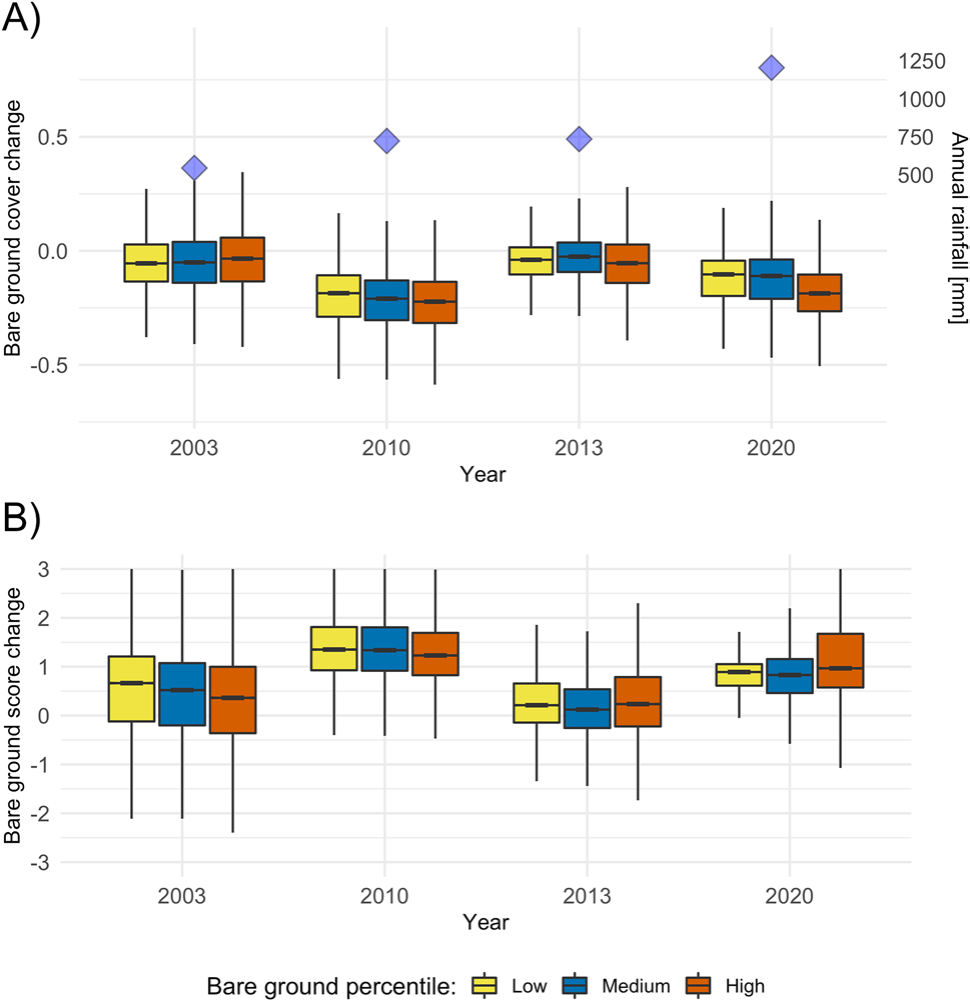
Recovery in bare ground scores following the four years of largest degradation. Split by bare ground percentile, based on the median degradation scores of the final three years of the time series (2018–2020). (**A**) *Absolute recovery*, positive numbers along the y axis signify an increase in bare ground, while negative numbers signify a decrease in bare ground. Blue diamonds indicate annual rainfall (November to October) based on CHIRPS version 2^54^ data. (**B**) *Relative recovery*, the proportion of decline that returns in the recovery year. A value of 1 = total recovery, 0 = no recovery, <0 = continued decline, >1 net improvement. Whiskers represent 95% confidence intervals around the median. Outliers are not shown. All contrasts were statistically different in the ANOVA tests.

**Figure 7:**
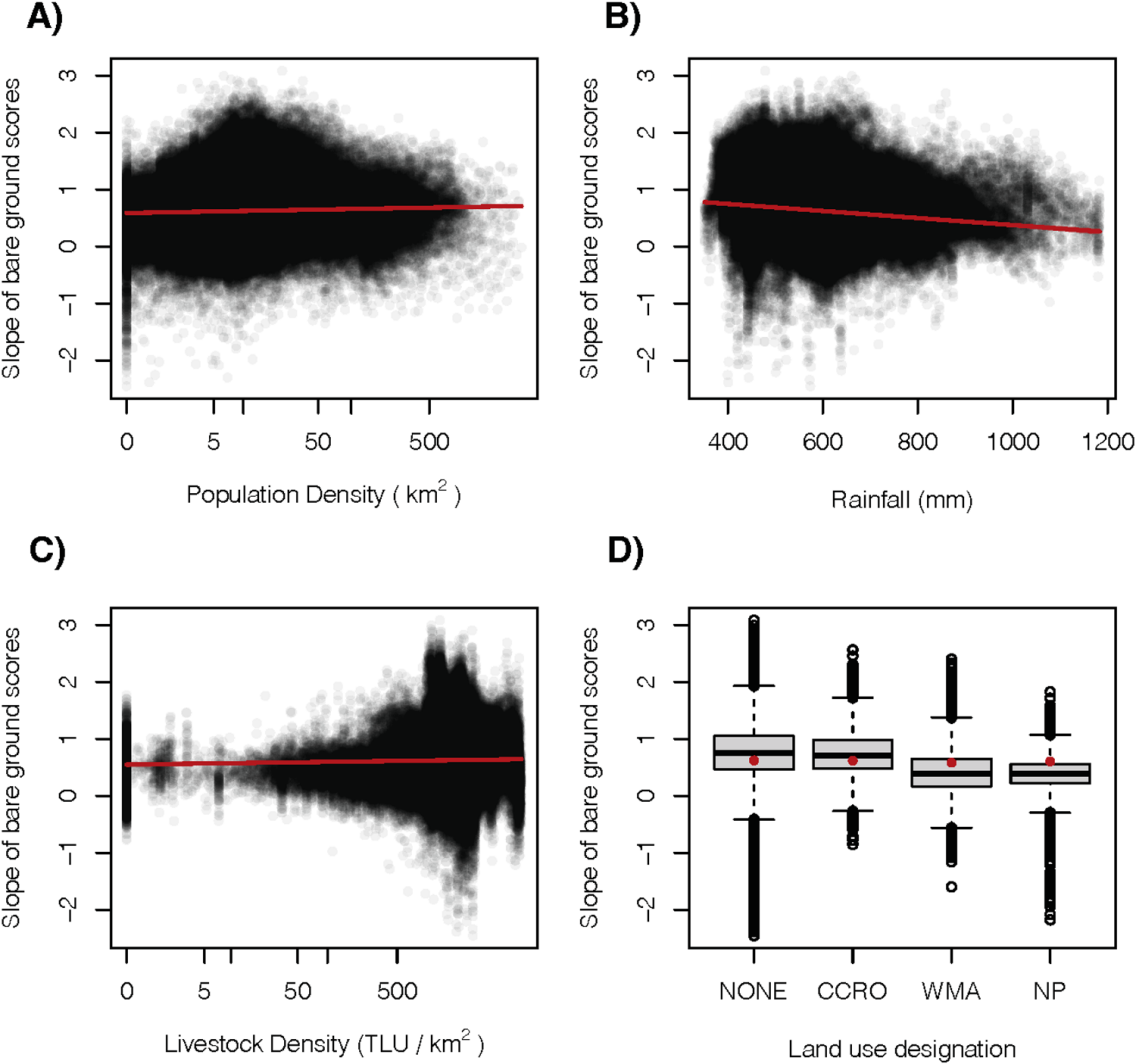
Effect plots showing the correlations between covariates and the rate of change in bare ground cover across the time period. (**A**) Human population density, (**B**) Mean annual rainfall, (**C**) Livestock density and (**D**) Land use designation.

Formal statistical analysis of the cell-specific change of bare ground scores between 2000 and 2020 showed significant effects of all covariates (Fig. 7, Table 2). We found evidence that mean annual rainfall had the strongest effect on the long-term trend of bare ground scores compared to the other covariates in the model, with drier areas showing steeper increases in bare ground scores than wetter areas (Fig. 7B). We found increases of bare ground scores over the last 20 years in all areas, independent of land use designation (Fig. 7, Table 2). The bare ground cover change in land that had been designated as CCROs by the end of the time period was no different from land in the NONE category, while national parks and WMAs had a lower rate of increase in bare ground than NONE (Fig. 7D). Both human population density and livestock density had small correlations with the change of bare ground scores, such that areas with higher human density, as well as more livestock, experienced increasing bare ground cover rates (Fig. 7A,C).

**Table 2:** Parameter estimates and their 95% credible intervals for INLA model results predicting the rate of bare ground scores over time. Positive estimates correspond with positive rates of bare ground scores, and negative estimates with negative rates of bare ground scores. Parameters with credible intervals that do not overlap zero (bold) may be considered well supported by these Bayesian models.

Parameter	Estimate	Credible intervals
Intercept	**0.621**	**(0.611, 0.631)**
Human population density	**0.015**	**(0.011, 0.015)**
Rainfall	**-0.052**	**(-0.059, -0.045)**
Livestock density	**0.015**	**(0.008, 0.023)**
Designation: CCRO	-0.004	(-0.017, 0.008)
Designation: NP	**-0.04**	**(-0.056, -0.025)**
Designation: WMA	**-0.018**	**(-0.028, -0.009)**

## Discussion

We found evidence that, in our study area, degradation seems to result primarily from a loss of resistance to change, not a lack of recovery. Land identified in the highest bare ground percentile by the end of the time period experienced slightly larger declines in condition in most years of widespread annual decline, but maintained recovery potential throughout. Absolute recovery in ultimately more degraded sites (i.e. sites in the highest bare ground percentile during the last three years of the time period) was actually slightly greater in all but the driest recovery year. Our results show that, as one shock rapidly follows another, sites that are ultimately degraded do not have time to fully recover between shocks. Sites with ultimately highest bare ground scores would have had to repeatedly exhibit net improvement to balance out the increased decline, but this degree of recovery only occurred during years of high rainfall. As we explicitly included annual rainfall in our models, we do not consider that large-scale climatic processes on the decadal scale can explain our overall findings. It should be noted that our estimated degradation scores exhibited significant variation, leading to small median effects. Nonetheless, we observed consistent patterns across years, suggesting that these trends go beyond statistical noise.

Although there is much in the ecological literature that defines separate concepts for resistance and recovery^25,26,81^, our results provide evidence that, in this area, repeated environmental shocks are a driver of bare ground cover. However, defining recovery will require more than measuring improvements in our bare ground index. The conceptualization of degradation as delivered through a repeated process of shock followed by partial recovery is important because it gives hope for eventual restoration of these rangelands. Although it seems unlikely that ecological shocks are to decline in frequency given global perturbations of the climate system^82,83^, the fact that recovery potential remains suggests that reducing factors that decrease resistance to change could allow rapid recovery. Indeed, the ability to recover quickly from year to year has long been at the core of traditional management of these rangelands: heavy use over a few years could lead to severe local degradation, but nomadic people moved away for a few years allowing natural recovery^84,85^. Today, such movements are increasingly restricted by fragmentation of rangelands, mainly through increasing agriculture^86,87^, rendering it necessary for pastoralists to remain in what may otherwise have been only temporarily degraded sites^88,89^, resulting in declining grassland productivity and increased degradation^20,90,91^.

Under the current prediction of overall increased rainfall in East Africa^83,92^, the increased recovery potential with higher rainfall could give hope for the eventual restoration of these rangelands. However, rainfall predictions for the region are complex. Historically, long season rainfall in East Africa has declined^93,94^. Future climate change is predicted to lead to more intense rainfall on individual days during the long rain season^83,95,96^ and increasing rainfall during the short rain season^96,97^, but also an increase in dry days^98^ and frequency and duration of droughts^34,99^. Overall, studies predict an increased frequency of high rainfall events associated with storms over Africa, linked to climate change^100,101^. It is currently unclear how these changing patterns will affect rangeland recovery potential. Due to the temporal resolution of our study, considering total annual rainfall only, we are lacking this insight into the finer scale rainfall patterns driving bare ground cover change. The clear association of rangeland recovery with total rainfall during our study period suggests that temporal variation in rainfall does not yet override the spatial effect of total annual rainfall, but the relationship with rainfall patterns should be explored to evaluate the future risk to rangeland recovery posed by climate change in East Africa. Furthermore, the spatial pattern of rainfall in relation to recovery should be considered. Rainfall in savanna grasslands is characterized by a high spatial patchiness^102^. Our study related recovery to rainfall averaged over the whole study area, disregarding local trends, and thus the finer scale responses to rain. Our results are applicable at the wider scale, but more research is needed to confirm the relationship between rainfall and recovery at the regional scale of management areas.

The general association of rangeland recovery with rainfall highlights a potential caveat of studies including bare ground as a parameter: While bare ground is the most visible expression of resistance and recovery on a wider landscape scale (enabling coarse satellite analyses), it is also potentially misleading, because of the high sensitivity to rainfall. Even rangeland well on its path to degradation (resulting from loss of resistance and/or recovery potential) may show a flush of growth following high rainfall events, leading to short-term variability of bare ground estimates. It is therefore crucial to account for rainfall, as it was done here: i) use rainfall to inform estimates of bare ground cover, ii) consider the long-term trends of bare ground, after factoring out temporal variation in rainfall, and iii) account for spatial variation in rainfall when analysing spatial patterns of bare ground cover. But even where rainfall is accounted for, the use of bare ground alone may present problems. In the context of recovery, for example, we registered a rangeland as ‘recovered’ if vegetation cover had sufficiently increased, even though this might be through fast-growing invasive or toxic species, rather than palatable grasses. For this bias to lead to false conclusions, however, such invasive and toxic plants would have to be more dominant than grazeable vegetation, during times of regrowth, across the hundreds of pixels considered for this study. Surveys suggest that the majority of these invasives are still low in numbers in the region^103^, although this might become a greater risk in the future.

We succeeded in generating classified maps of bare ground scores from Landsat images since 2000. Formal validation confirmed pixel-level correlations between observed and predicted test regions for both parameters of interest, bare ground and ITP, but further evaluation based on expected year-to-year variation only validated predictions of bare ground. Using satellite imagery to identify specific vegetation types, particularly at a taxonomic level, has long been a challenge in the field of remote sensing, requiring data at higher resolution than used in this study^65,66^. With the establishment of the Sentinel-2 program, providing imagery with high spatial (10 m) and temporal (5-day) resolution^104^, researchers have increasingly overcome this challenge, particularly through the use of time series analyses, utilizing images at peak vegetation intensity^66,105^. However, data are not available before 2015, making these data unsuitable for the analysis of longer term trends, as are expected in savanna habitats. The final generated landscape level patterns of bare ground cover in our study were consistent with known land use patterns. Our bare ground scores showed considerable variation year to year, which is consistent with known patterns of inter-annual variation in grass productivity in semi-arid savannas^106,107^. We found evidence for increases in bare ground scores across the study area, particularly in the driest areas, exactly as reported elsewhere^108–110^, and in line with the large scale increases in bare ground observed in East African grasslands over the last two decades^24^.

We found the expected positive relationship between human density and degradation^22,108^. Furthermore, we found that increases in bare ground cover were positively correlated with livestock density. While this result corroborates the negative relationship between high grazing intensity and grass biomass observed in African savannas^20^, the relatively low spatial resolution of the FAO product used to estimate livestock density^77^ lends only limited interpretability to this finding. Under the current predictions of continued growth in human population and demand for livestock products, however, this potential pressure on rangelands is unlikely to decrease. The signs of lower increases of bare ground cover in WMAs and national parks point to their effectiveness in mitigating large scale declines. Finally, we found no evidence for reduced bare ground increases in land that became CCROs. CCROs are a relatively new tool being promoted to enable the effective management of rangelands by local communities^111–113^, so it may seem surprising that these areas do not show improvements during the final years, especially as our analysis accounts for differences in rainfall and human population density that may differ between sites. In practice, however, CCROs are not in themselves a solution to the problem of degradation: although they establish areas dedicated for grazing, they do not yet provide sufficient management guidance around that grazing^111^. Consequently, once established they may generate grazing honeypots that increase degradation locally, rather than resolve the problems associated with poor land use. However, several CCROs are now engaged in sustainable grazing and management schemes, which might lead to a future reduction of degradation in these areas. Our results show that the ability of these sites to recover if effectively managed is undiminished, which speaks to the potential effectiveness of sustainable management schemes in CCROs. Establishing responsible community management may well be the first step that is needed if degradation is to be reduced. Continued monitoring of rangeland conditions in these areas is needed to establish the effectiveness of these new management strategies. It should be added that the list of CCROs included for the study area is not exhaustive: Due to the time-consuming process involved in establishing these areas, not all boundaries were available at the time of analysis.

Future research should include measurements of finer-scale qualities of rangeland health, to overcome some of the caveats highlighted in this study. Such measurements could include species composition of the vegetation cover, plant traits related to palatability, individual resistance and recovery capacity of plants, or soil properties (e.g. composition, compaction). The latter could provide important insights into the mechanisms of grassland degradation and recovery at a finer spatial scale, given its influence on water retention^114^, soil erosion^115,116^, and soil microbial activity^117^. Additional management techniques could be considered, such as the frequency of fires, an important historic and contemporary management strategy in the region^118^. These variables may reveal a more complete picture of the pathways to degradation, and enable more effective rangeland management strategies. If the qualities underlying rangelands resistant to degradation are identified, they could be targeted in order to further promote rangeland resistance in the face of greater shocks and stress.

## Supplementary Information

Supplementary Information.

## Data Availability

The code used in Google Earth Engine can be accessed online using the following link: https://anonymous.4open.science/r/GEE_code_Wiethase_et_al-2246. R code for the data analysis can be accessed online using the following link: https://github.com/jwiethase/Wiethase-Scientific-Reports-2022.
