# Supplementary material for: Pathways of degradation in rangelands in Northern Tanzania show their loss of resistance, but potential for recovery
Source: Sci Rep. 2023 Feb 22;13:2417. doi: 10.1038/s41598-023-29358-6 (PMC9946995; doi:10.1038/s41598-023-29358-6)
Supplement: Supplementary file 1 — Supplementary Information. [file 41598_2023_29358_MOESM1_ESM.pdf]

Supplementary material for the manuscript titled: Pathways of degradation in rangelands in Northern Tanzania show their loss of resistance, but potential for recovery

Joris H. Wiethase[1,*,+], Rob Critchlow[1,+], Charles Foley[2], Lara Foley[2], Elliot J. Kinsey[3], Brenda G. Bergman[3], Boniface Osujaki[4], Zawadi Mbwambo[5], Paul Baran Kirway[4], Kelly R. Redeker[1], Susan E. Hartley[6], and Colin M. Beale[1,7]

[1] *University of York, Department of Biology, York, YO10 5DD, GB*
[2] *Lincoln Park Zoo, Tanzania Conservation Research Program, Chicago, Illinois, 60614, US*
[3] *The Nature Conservancy, Arlington, Virginia, 22203, US*
[4] *Independent contributor*
[5] *Zoological Society of London, Tanzania Program, London, NW1 4RY, GB*
[6] *University of Sheffield, School of Biosciences, Sheffield, S10 2TN, GB*
[7] *University of York, York Environmental Sustainability Institute, York, YO10 5DD, GB*
[*] *jhw538@york.ac.uk*
[+] *these authors contributed equally to this work*

ABSTRACT

Semiarid rangelands are identified as at high risk of degradation due to anthropogenic pressure and climate change. Through tracking timelines of degradation we aimed to identify whether degradation results from a loss of resistance to environmental shocks, or loss of recovery, both of which are important prerequisites for restoration. Here we combined extensive field surveys with remote sensing data to explore whether long-term changes in grazing potential demonstrate loss of resistance (ability to maintain function despite pressure) or loss of recovery (ability to recover following shocks). To monitor degradation, we created a bare ground index: a measure of grazeable vegetation cover visible in satellite imagery, allowing for machine learning based image classification. We found that locations that ended up the most degraded tended to decline in condition more during years of widespread degradation but maintained their recovery potential. These results suggest that resilience in rangelands is lost through declines in resistance, rather than loss of recovery potential. We show that the long-term rate of degradation correlates negatively with rainfall and positively with human population and livestock density, and conclude that sensitive land and grazing management could enable restoration of degraded landscapes, given their retained ability to recover.

1 Supplementary Tables

Table 1. Predictor variables used as input for the svr classifier. *L7* landsat 7 product, *L8* landsat 8 product, *B1-7* band number in landsat 7 or 8 products. GEE: Google Earth Engine.

Predictor	Notes
blue	0.45 - 0.52 µm (L7, B1), 0.45 - 0.51 µm (L8, B2)
green	0.52 - 0.60 µm (L7, B2), 0.53 - 0.59 µm (L8, B3)
red	0.63 - 0.69 µm (L7, B3), 0.64 - 0.67 µm (L8, B4)
nir	0.77 - 0.90 µm (L7, B4), 0.85 - 0.88 µm (L8, B5)
swir1	1.55 - 1.75 µm (L7, B5), 1.57 - 1.65 µm (L8, B6)
swir2	2.09 - 2.35 µm (L7, B7), 2.11 - 2.29 µm (L8, B7)
EVI	$\frac{2.5\times NIR-Red}{NIR+6\times Red-7.5\times Blue+1}$
BSI	$\frac{(SWIR1-Red)-(NIR-Blue)}{(SWIR1+Red)+(NIR+Blue)}$
MSAVI	$\frac{(2\times NIR+1-\sqrt{(2\times NIR+1)^2-8\times(NIR-Red)})}{2}$
EVI, BSI, MSAVI magnitude	cos.hypot(sin).multiply(5) (GEE code)
EVI, BSI, MSAVI phase	sin.atan2(cos).unitScale(-Math.PI, Math.PI) (GEE code)
EVI, BSI, MSAVI val	harmonic_withVar.select(variable).reduce('mean') (GEE code)
CHIRPS total rainfall	May previous year to April of prediction year

Table 2. Data layers used in creating the rangeland-only mask.

Variable	Threshold	Resolution (Ref. year)	Source (ref.)
Surface water	Occurrence > 0	30 m (1984-2019)	GEE: "JRC/GSW1_2/GlobalSurfaceWater" ([1])
Rivers	Permanent water label	90 m (NA)	GEE: "MERIT/Hydro/v1_0_1" ([2])
Steep areas	Slope > 45°	30 m (2000)	GEE: "USGS/SRTMGL1_003" ([3])
Urban areas	Urban >= 50%	100 m (2019)	GEE: "COPERNICUS/Landcover/100m/Proba-V/Global" ([4])
Known crops	Cropland label	30 m (2015)	https://lpdaac.usgs.gov/products/gfsad30afcev001/ ([5])
Forests	Tree cover > 50%	30 m (2000-19)	GEE: "UMD/hansen/global_forest_change_2019_v1_7" ([6])
High elevation	Elevation > 2500 m	30 m (NA)	GEE: "USGS/SRTMGL1_003" ([3])

Table 3. Total area covered (including non-rangeland), and number of pixels used in the land use analysis in the different land use designation sites considered in the study. NP: National Park (NP), WMA: Wildlife Management Area, CCRO: Certificate of Customary Right of Occupancy, NONE: No official management/protection scheme.

Land use designation	Total area (km$^{2)}$	Number of pixels
NP	2944.57	62274
WMA	6715.98	156306
CCRO	1252.27	29979
NONE	20295.9	427246

2 Supplementary Figures

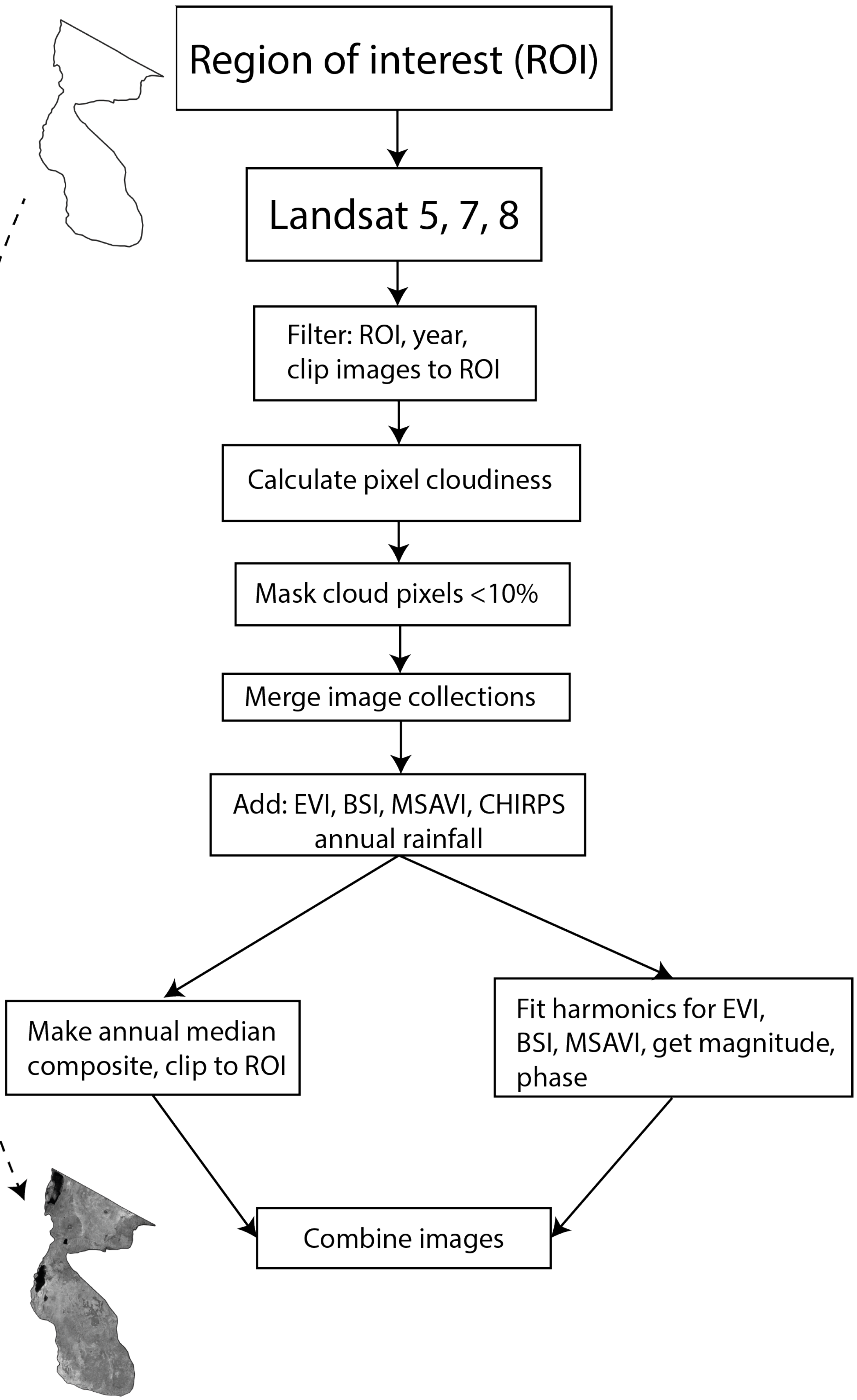

Figure 1. Conceptual overview of the steps involved in creating annual composite maps for the study area.

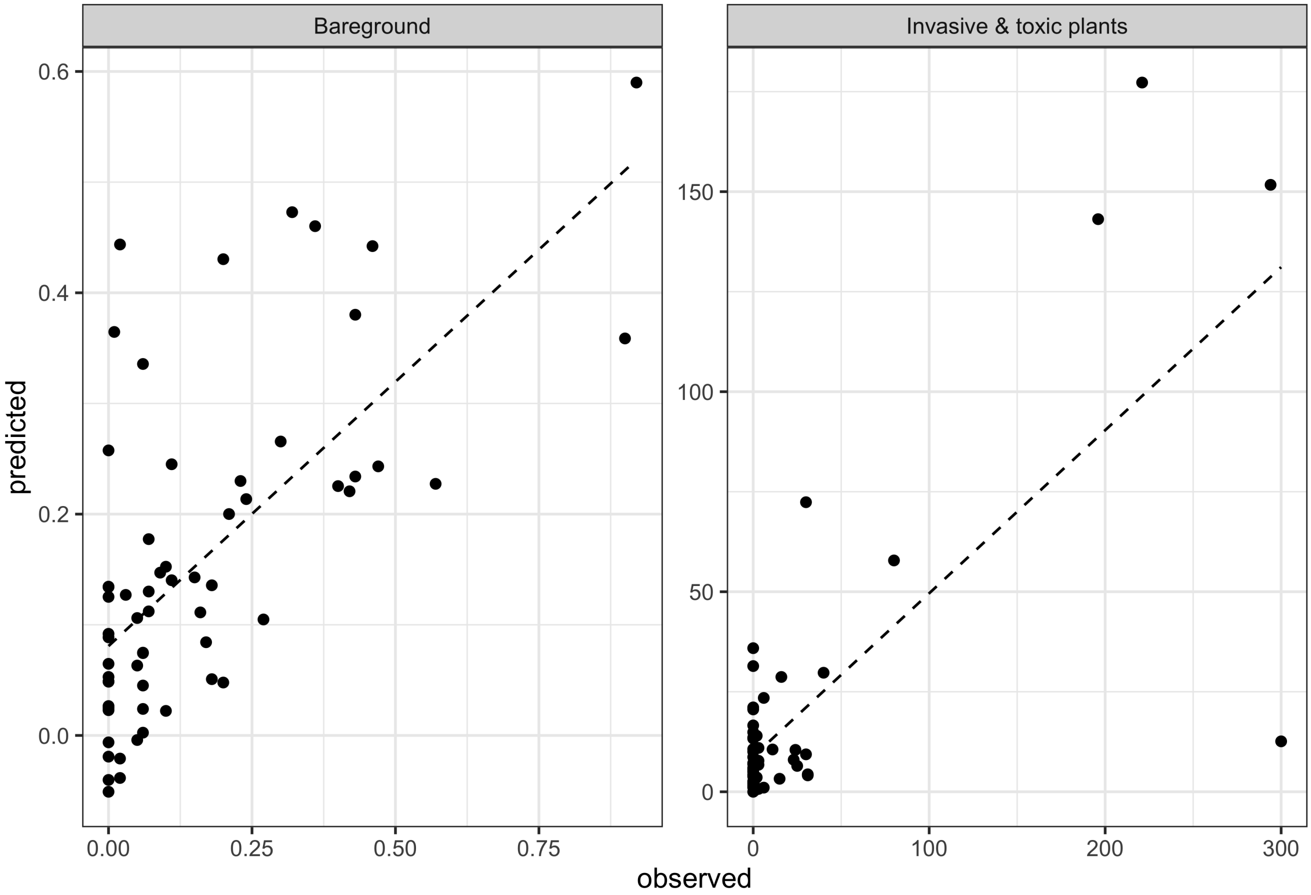

Figure 2. Validation of degradation scores, observed vs. predicted on 25% of sites not included in modelling for bare ground and number of invasive & toxic plants. Based on the final model, fine-tuned during cross-validation. Dashed lines are trend lines from a linear model on the data.

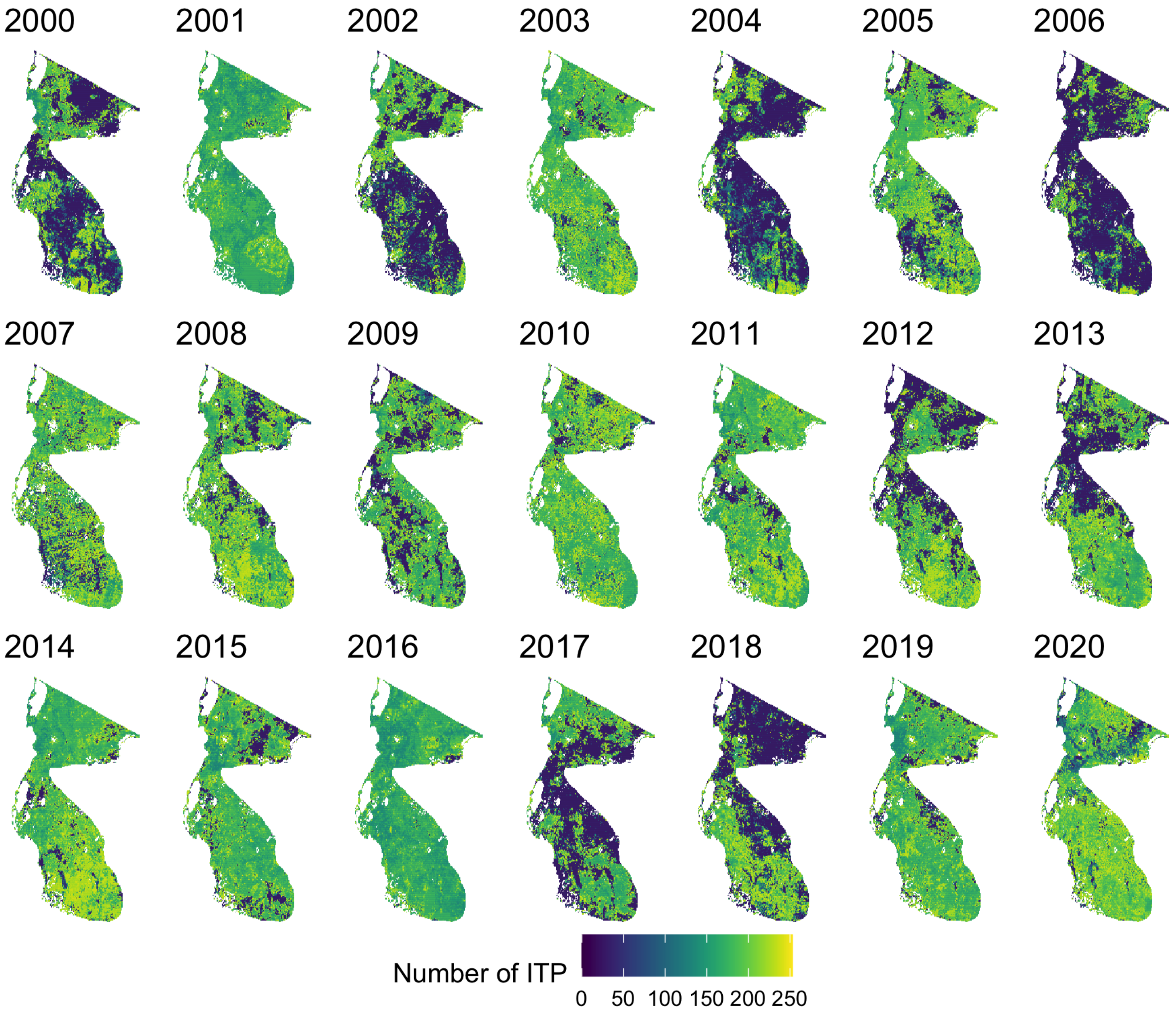

Figure 3. Yearly maps of invasive & toxic plant (ITP) cover, based on predictions from the machine learning model. Darker colors correspond to lower ITP cover. The maps were created using R 3.2.2[7].

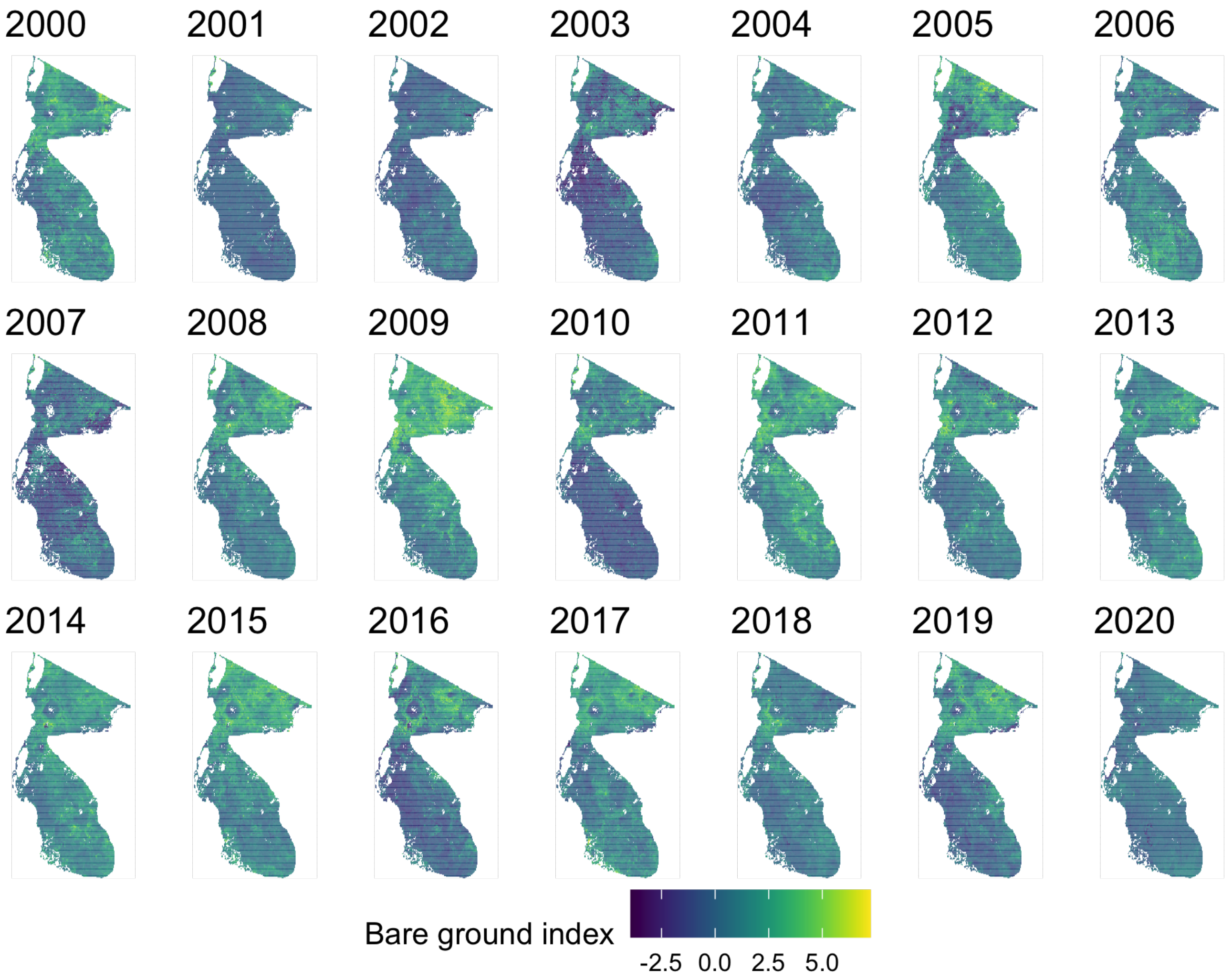

Figure 4. Yearly maps of bare ground index, based on predictions from the machine learning model, not normalized. Darker colors correspond to a lower bare ground index. The maps were created using R 3.2.2[7].

References

1. Pekel, J.-F., Cottam, A., Gorelick, N. & Belward, A. S. High-resolution mapping of global surface water and its long-term changes. *Nature* **540**, 418–422 (2016).
2. Yamazaki, D. *et al.* MERIT hydro: A High-Resolution global hydrography map based on latest topography dataset. *Water Resour. Res.* **55**, 5053–5073 (2019).
3. Farr, T. G. *et al.* The shuttle radar topography mission. *Rev. Geophys.* **45**, RG2004 (2007).
4. Buchhorn, M. *et al.* Copernicus global land cover Layers—Collection 2. *Remote. Sens.* **12**, 1044 (2020).
5. Xiong, J. *et al.* NASA making earth system data records for use in research environments (MEaSUREs) global food security-support analysis data (GFSAD) cropland extent 2015 africa 30 m V001. (2017).
6. Hansen, M. C. *et al.* High-Resolution global maps of 21st-century forest cover change. *Science* **342**, 850–853 (2013).
7. R Core Team. R: A language and environment for statistical computing. *R Foundation for Stat. Comput. Vienna, Austria.* (2016).
